# Why we should insist on education about and implementation of ultrasound-guided regional anesthesia in intensive care units

**DOI:** 10.3325/cmj.2025.66.231

**Published:** 2025-06

**Authors:** Katarina Tomulić Brusich, Mirna Dubrović, Miroslav Župčić

**Affiliations:** 1 Department of Anesthesiology, Intensive Care and Pain Medicine, Clinical Hospital Centre Rijeka, Rijeka, Croatia; 2 Department of Anesthesiology, Resuscitation, Emergency and Intensive Care Medicine, Faculty of Medicine, University of Rijeka, Rijeka, Croatia; *katarinatb@uniri.hr*; 3 Department of Clinical Medical Sciences II, Faculty of Health Studies, University of Rijeka, Rijeka, Croatia.

Patients in the intensive care unit (ICU) often experience moderate to severe pain attributable to pre-existing conditions, surgical interventions, or routine procedures. Insufficient pain treatment can adversely affect various organ systems, extending ICU durations and elevating mortality rates (1). The cardiovascular system is most affected, with patients demonstrating tachycardia and elevated myocardial oxygen demand. In non-cardiac surgical patients, postoperative pain is correlated with myocardial damage, compromised respiratory function, ileus, thrombosis, impaired wound healing, and heightened infection risk (2,3). Moreover, inadequately managed acute pain may promote chronic pain onset. The rates of persistent pain range from 33% to 56% six months to two years after ICU discharge (4,5). Despite the awareness of these serious consequences, many ICUs struggle with proper pain assessment and treatment.

Opioids are still considered the cornerstone for managing pain in the ICU. However, caution is advised due to the associated risks such as tolerance, withdrawal symptoms, the rise in ileus and delirium incidence, and respiratory depression (6). Non-opioid analgesics are also administered, specifically non-steroidal anti-inflammatory drugs and paracetamol, despite their possible adverse effects, such as renal compromise, particularly in critically ill patients. Furthermore, patients with Guillain-Barré syndrome and multiple sclerosis require the use of neuropathic pain therapeutics gabapentinoids (7).

Pain in the ICU is often assessed with standardized scales (visual or numerical analogue scales). However, most critically ill patients cannot communicate the level of experienced pain. For individualized pain management regimens, it is necessary to combine pharmacological and non-pharmacological strategies to achieve the best patient outcomes. Regular pain assessments, individualized titration of analgesics, and a multimodal approach are crucial in this respect (8-10). Nevertheless, the lack of uniform implementation guidelines across ICUs may make the application of these findings in practice challenging, and further research is needed. Additionally, biases may arise from relying solely on the available literature, which might not fully encompass all variables present in various ICU settings (11,12).

Recently, opioids use has been increasingly replaced by regional anesthesia (RA) techniques to alleviate pain while minimizing the associated side effects. RA provides excellent pain relief and reduces the stress response associated with surgical and critical illness (13). Reducing opioid use mitigates the occurrence of opioid-related adverse effects, such as sedation and delirium, enhances gut motility and splanchnic perfusion, and mitigates progression to chronic pain states. All these factors may promote early ambulation and reduction of mechanical ventilation duration (14). RA techniques such as thoracic epidural analgesia (TEA) have shown effectiveness in rib fractures, acute pancreatitis, and complex regional pain syndrome, in which traditional systemic analgesics have been proven inadequate. TEA can tangibly improve patient outcomes in terms of a reduced need for vasopressors and a shorter ICU stay (15). However, concerns regarding infection, coagulopathy, and patient consent can limit the use of these techniques.

Conversely, peripheral nerve blocks (PNBs) and fascial plane blocks (FPBs) may be beneficial in critically ill patients concerned about the potential side effects of neuraxial procedures, as these techniques are commonly employed in perioperative contexts. Thoracic or abdominal PNBs and FPBs for acute pain management are of particular interest to critical care physicians within the framework of multimodal analgesia. The concept of PNB for analgesia in the upper or lower limbs requires the administration of a local anesthetic, with or without adjuvants, close to the major nerves. FPBs are methods of regional anesthesia that target the space between two distinct fascial layers for needle insertion and injection. Analgesia is primarily achieved through the distribution of local anesthetics to the nerves located within this plane and the surrounding tissues. Thoracic FPBs (including transversus abdominis plane blocks, quadratus lumborum blocks, rectus sheath blocks, paravertebral blocks, and parasternal blocks) and abdominal wall FPBs (including transversus abdominis plane blocks, rectus sheath blocks, and subcostal tap blocks) constitute valuable enhancements to various of pain-relief methods. Ultrasound-guided RA is particularly advantageous in the ICU setting. A direct view of nerves and adjacent anatomy, continuous monitoring of the needle tip and local anesthetic dispersion, along with the capacity to detect early intraneural and intravascular injections, mitigate problems and enhance safety (16).

## Specific concerns for regional anesthesia in critically ill ICU patients

Critically ill patients exhibit distinct complications in contrast to healthy people undergoing a perioperative PNB/FPB. In sedated individuals, identifying potential concerns may prove more difficult, which complicates the monitoring procedure (17). Additionally, it may be challenging to position patients appropriately for optimal access. The factors that additionally complicate regional anesthetic treatments are tissue edema, the presence of multiple catheters, and hemodynamic or bleeding-related fluctuations. Over the last ten years, ultrasound-guided (USG) approaches have emerged as a standard practice for regional anesthetic procedures, offering enhanced advantages for ICU patients, particularly in complex anatomical conditions (18). Ultrasound guidance techniques are invaluable, particularly in contrast to nerve stimulation guidance, which may be diminished by neuromuscular impairment in severely ill patients (19).

The condition of critically ill patients often affects pharmacokinetics and pharmacodynamics in various ways by changing the volume of distribution and drug metabolism. Hepatic or renal insufficiency may influence the clearance of local anesthetics, potentially increasing the risk of local anesthetic systemic toxicity (LAST) (20). Once again, USG techniques allow accessible, secure, and accurate anatomical structure identification, facilitating the administration of reduced doses of local anesthetics to decrease the risk of LAST.

Most critically ill patients receive anticoagulant drugs as a standard treatment to avert deep vein thrombosis, which restricts the use of RA procedures. Therefore, RA administration needs to be carefully coordinated with anticoagulant administration to reduce the risk of hematomas and adhere to established guidelines for RA in patients receiving anticoagulants (21). Nevertheless, in the presence of specific issues and contraindications associated with neuraxial treatments, PNBs and FPBs may present a beneficial option for ICU patients when neuraxial techniques are deemed unsuitable or hazardous. USG is additionally advantageous in this scenario.

Another consideration when evaluating the benefits of peripheral nerve blocks over neuroaxial anesthesia in the ICU is the risk of infection associated with mechanical ventilation. Mechanical ventilation modifies intrathoracic pressures, and patient positioning alters the distribution of local anesthetic in the epidural or spinal space, which all complicate the management of neuroaxial techniques (22). The incidence of patients with sepsis is markedly elevated in the ICU compared with other units. These patients are at risk from catheter-associated infections, which prompts inquiries over the safety of supplementary invasive procedures and/or the ongoing use of RA catheters (23). PNBs and FPBs are regarded as beneficial in this context.

## Limitations of regional anesthesia in the ICU

Most of the current information on RA comes from surgical patients and perioperative settings. The majority of RA techniques in the ICU are limited to cases of rib fracture or acute pancreatitis treatment (20). There is a shortage of randomized controlled studies investigating the application of RA in the ICU, which leaves some of the conclusions less robust (11). There is a need for further research to develop clear guidelines for a safe application of RA across diverse patient populations, focusing especially on those with complex underlying conditions, such as frail patients who exhibit diminished cardiorespiratory reserve and those undergoing long-term therapies that affect hemostasis.

The prevailing trend among health care providers is to advocate for RA to enhance pain treatment in the ICU, consequently diminishing opioid consumption. However, there are concerns about the consistency of practice and training in RA techniques among the ICU staff. Recent improvements in the ease of using ultrasound-guided PNB and FPB have increased the frequency of use of RA, providing significant benefits when combined with these popular multimodal approaches (16). Many of these blocks are inherently safer than neuroaxial techniques while simultaneously substantially contributing to pain management. However, challenges remain regarding the consistency of practice and training in RA techniques among ICU staff.

In conclusion, the application of ultrasonography for RA procedures appears to be particularly advantageous in critically ill patients. The enhanced risk-benefit ratio renders PNBs/FPBs essential instruments for ICU physicians, particularly when neuraxial anesthesia is undesirable or contraindicated. This is the primary rationale for implementing RA in the ICU setting. Likewise, we should insist on education in the domain of ultrasound-guided RA, from the early stages of residency to senior staff. Furthermore, future research ought to investigate the role of peripheral RA procedures in critically ill patients, which could lead to standardized training protocols for ICU staff.
